# Dibromido{2-hydr­oxy-*N*′-[phen­yl(2-pyrid­yl)methyl­ene]benzohydrazide}copper(II)

**DOI:** 10.1107/S1600536809038070

**Published:** 2009-09-26

**Authors:** Ling-Qian Kong, Xiu-Ping Ju, Da-Cheng Li

**Affiliations:** a Dongchang College of Liaocheng University, Shandong 25200, People’s Republic of China; b College of Chemistry and Chemical Engineering, Liaocheng University, Shandong 252059, People’s Republic of China

## Abstract

In the title complex, [CuBr_2_(C_19_H_15_N_3_O_2_)], the metal ion is coordinated by the *N*,*N*′,*O*-tridentate 2-hydr­oxy-*N*′-[phen­yl(2-pyrid­yl)methyl­ene]benzohydrazide ligand and two bromide ions, resulting in a distorted CuN_2_OBr_2_ square-based pyramidal coordination geometry with one bromide ion in the apical site. An intra­molecular N—H⋯O hydrogen bond occurs in the ligand. In the crystal, mol­ecules are connected by inter­molecular C—H⋯O, C—H⋯Br and O—H⋯Br inter­actions.

## Related literature

For the crystal structures of metal complexes with 2-benzoyl­pyridine salicyloylhydrazone, see: Sur *et al.* (1993 ▶); Seth & Chakraborty (1984 ▶); Dan *et al.* (1989 ▶).
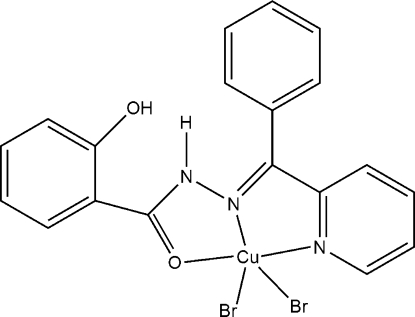


## Experimental

### 

#### Crystal data

[CuBr_2_(C_19_H_15_N_3_O_2_)]
*M* *_r_* = 540.70
Monoclinic, 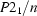

*a* = 8.0779 (11) Å
*b* = 16.302 (2) Å
*c* = 15.0376 (18) Å
β = 97.624 (2)°
*V* = 1962.8 (4) Å^3^
*Z* = 4
Mo *K*α radiation
μ = 5.20 mm^−1^
*T* = 298 K
0.23 × 0.19 × 0.15 mm

#### Data collection

Bruker SMART CCD diffractometer
Absorption correction: multi-scan (*SADABS*; Bruker, 2003 ▶) *T*_min_ = 0.381, *T*_max_ = 0.509
8676 measured reflections
3446 independent reflections
2426 reflections with *I* > 2σ(*I*)
*R*_int_ = 0.034

#### Refinement

*R*[*F* ^2^ > 2σ(*F* ^2^)] = 0.034
*wR*(*F* ^2^) = 0.082
*S* = 1.01
3446 reflections
244 parameters
H-atom parameters constrained
Δρ_max_ = 0.94 e Å^−3^
Δρ_min_ = −0.58 e Å^−3^

### 

Data collection: *SMART* (Bruker, 2003 ▶); cell refinement: *SAINT* (Bruker, 2003 ▶); data reduction: *SAINT*; program(s) used to solve structure: *SHELXS97* (Sheldrick, 2008 ▶); program(s) used to refine structure: *SHELXL97* (Sheldrick, 2008 ▶); molecular graphics: *SHELXTL* (Sheldrick, 2008 ▶); software used to prepare material for publication: *SHELXTL*.

## Supplementary Material

Crystal structure: contains datablocks I, global. DOI: 10.1107/S1600536809038070/hb5107sup1.cif

Structure factors: contains datablocks I. DOI: 10.1107/S1600536809038070/hb5107Isup2.hkl

Additional supplementary materials:  crystallographic information; 3D view; checkCIF report

## Figures and Tables

**Table 1:** Selected bond lengths (Å)

Cu1—N2	1.966 (3)
Cu1—N3	2.018 (3)
Cu1—O1	2.083 (2)
Cu1—Br1	2.3469 (6)
Cu1—Br2	2.5931 (8)

**Table 2:** Hydrogen-bond geometry (Å, °)

*D*—H⋯*A*	*D*—H	H⋯*A*	*D*⋯*A*	*D*—H⋯*A*
N1—H1⋯O2	0.86	1.92	2.574 (4)	131
O2—H2⋯Br2^i^	0.82	2.35	3.153 (3)	166
C11—H11⋯O1^ii^	0.93	2.58	3.503 (5)	170
C10—H10⋯Br1^ii^	0.93	2.81	3.575 (4)	141
C15—H15⋯Br2^iii^	0.93	2.82	3.742 (4)	171

Symmetry codes: (i) 
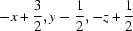
; (ii) 
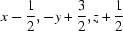
; (iii) 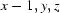.

**Fig. 1.**
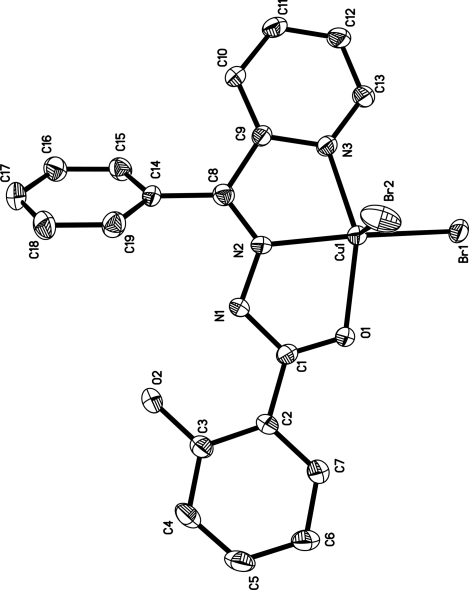
The molecular structure of (I) showing 30% displacement ellipsoids. C-bound H atoms have been omitted for clarity.

## supplementary crystallographic information

### Comment

A large number of salicyloylhydrazone complexes have been reported and studied. However, the metal complexes of 2-benzoylpyridine salicyloylhydrazone reported are limited to Zn (Sur *et al.*, 1993), Ni (Seth *et al.*, 1984) and (Dan *et al.*, 1989). Here, we have synthesized and will report a new 2-benzoylpyridine salicyloylhydrazone complex Cu_(_C_19_H_15_N_3_O_2_)Br_2_, which was was characterized by X-ray diffraction and elemental analysis. The crystals suitable for X-ray diffraction studies were obtained by slow evaporation of the mother liquid. In this paper, we will display the crystal structure of the title complex.

The title complex(Fig.1), Cu_(_C_19_H_15_N_3_O_2_)Br_2_ is composed of a Cu atom, a 2-benzoylpyridine salicyloylhydrazone ligand molecule and two bromines. The ligand is bound to Cu atom by a carbonyl O, a pyridine N and a hydrazone N to form two juxtaposed five-membered chelate rings. Cu lies in a five-coordinated and square-pyramid coordination geometry with the _O_N_2_Br_2_ set of donor atoms. The equatorial coordination sits are occupied by O1, N2, N3, Br1 and the axial coordination atom is Br2 with the distance of Cu1—Br2 2.5931 (8) A. In the structure, there are intramolecular N—H···O interactions. Except that, the complex is linked into one-dimensional chain by intermolecular C—H···Br interactions, and the neighboring chains form a two-dimensional network structure *via* C—H···Br and O—H···Br interactions. A three-dimensional network structure is connected *via* C—H···O and C—H···Br interactions between adjacent two-dimensional networks. So the complex is linked into a three-dimensional network structure *via* intermolecular C—H···O, C—H···Br and O—H···Br interactions.

### Experimental

CuBr_2_.H_2_O (0.25 mmol 0.065 g) was dissolved in 10 ml MeOH and a 10 ml 1,1-dichlorinemethane solution of 2-benzoylpyridine salicyloylhydrazone (0.25 mmol 0.080 g) was added dropwise to the former. The mixture was stirred for six hours until the solution color became dark green.The dark green solution was stirred for five hours and filtered. The filtrate layered with Et_2_O to resulted in dark green blocks of (I) at room temperature. m.p.>573 K. Elemental analysis for _C_19_H_15_Cu_N_3_O_2_Br_2_ calculated: C 42.21, H 2.80 N 7.77%; found: C 42.32, H 2.54, N 7.68%.

### Refinement

All H atoms were placed geometrically and treated as riding on their parent atoms with C—H = 0.93 Å, O—H = 0.82Å and N—H = 0.86Å [*U*_iso_(H) = 1.2*U*_eq_(carrier)].

### Crystal data

**Table:**

[CuBr_2_(C_19_H_15_N_3_O_2_)]	*F*(000) = 1060
*M**_r_* = 540.70	*D*_x_ = 1.830 Mg m^−^^3^
Monoclinic, *P*2_1_/*n*	Mo *K*α radiation, λ = 0.71073 Å
Hall symbol: -P 2yn	Cell parameters from 2246 reflections
*a* = 8.0779 (11) Å	θ = 2.5–25.1°
*b* = 16.302 (2) Å	µ = 5.20 mm^−^^1^
*c* = 15.0376 (18) Å	*T* = 298 K
β = 97.624 (2)°	Block, dark green
*V* = 1962.8 (4) Å^3^	0.23 × 0.19 × 0.15 mm
*Z* = 4	

### Data collection

**Table:**

Bruker SMART CCD diffractometer	3446 independent reflections
Radiation source: fine-focus sealed tube	2426 reflections with *I* > 2σ(*I*)
graphite	*R*_int_ = 0.034
ω scans	θ_max_ = 25.0°, θ_min_ = 1.9°
Absorption correction: multi-scan (*SADABS*; Bruker, 2003)	*h* = −9→9
*T*_min_ = 0.381, *T*_max_ = 0.509	*k* = −17→19
8676 measured reflections	*l* = −17→11

### Refinement

**Table:**

Refinement on *F*^2^	Primary atom site location: structure-invariant direct methods
Least-squares matrix: full	Secondary atom site location: difference Fourier map
*R*[*F*^2^ > 2σ(*F*^2^)] = 0.034	Hydrogen site location: inferred from neighbouring sites
*wR*(*F*^2^) = 0.082	H-atom parameters constrained
*S* = 1.01	*w* = 1/[σ^2^(*F*_o_^2^) + (0.0396*P*)^2^] where *P* = (*F*_o_^2^ + 2*F*_c_^2^)/3
3446 reflections	(Δ/σ)_max_ = 0.001
244 parameters	Δρ_max_ = 0.94 e Å^−^^3^
0 restraints	Δρ_min_ = −0.58 e Å^−^^3^

### Special details

**Table:**

Geometry. All e.s.d.'s (except the e.s.d. in the dihedral angle between two l.s. planes) are estimated using the full covariance matrix. The cell e.s.d.'s are taken into account individually in the estimation of e.s.d.'s in distances, angles and torsion angles; correlations between e.s.d.'s in cell parameters are only used when they are defined by crystal symmetry. An approximate (isotropic) treatment of cell e.s.d.'s is used for estimating e.s.d.'s involving l.s. planes.
Refinement. Refinement of *F*^2^ against ALL reflections. The weighted *R*-factor *wR* and goodness of fit *S* are based on *F*^2^, conventional *R*-factors *R* are based on *F*, with *F* set to zero for negative *F*^2^. The threshold expression of *F*^2^ > σ(*F*^2^) is used only for calculating *R*-factors(gt) *etc*. and is not relevant to the choice of reflections for refinement. *R*-factors based on *F*^2^ are statistically about twice as large as those based on *F*, and *R*- factors based on ALL data will be even larger.

### Fractional atomic coordinates and isotropic or equivalent isotropic displacement parameters (Å^2^)

**Table:**

	*x*	*y*	*z*	*U*_iso_*/*U*_eq_	
Cu1	0.63317 (6)	0.70295 (3)	0.15437 (3)	0.03541 (16)	
Br1	0.63665 (6)	0.81344 (2)	0.05459 (3)	0.04420 (15)	
Br2	0.92790 (7)	0.70189 (3)	0.24686 (4)	0.06244 (18)	
N1	0.5874 (4)	0.53300 (18)	0.1785 (2)	0.0390 (9)	
H1	0.5667	0.4870	0.2027	0.047*	
N2	0.5516 (4)	0.60651 (17)	0.2140 (2)	0.0349 (8)	
N3	0.5009 (4)	0.75634 (19)	0.2434 (2)	0.0347 (8)	
O1	0.6872 (4)	0.60672 (14)	0.07174 (18)	0.0389 (7)	
O2	0.5854 (4)	0.37576 (16)	0.1626 (2)	0.0585 (10)	
H2	0.5800	0.3273	0.1765	0.088*	
C1	0.6586 (5)	0.5380 (2)	0.1020 (3)	0.0337 (10)	
C2	0.7003 (5)	0.4611 (2)	0.0589 (3)	0.0341 (10)	
C3	0.6649 (5)	0.3826 (2)	0.0886 (3)	0.0392 (11)	
C4	0.7099 (6)	0.3140 (2)	0.0427 (3)	0.0481 (12)	
H4	0.6868	0.2618	0.0625	0.058*	
C5	0.7887 (6)	0.3237 (3)	−0.0321 (3)	0.0523 (13)	
H5	0.8161	0.2774	−0.0631	0.063*	
C6	0.8283 (6)	0.3997 (3)	−0.0622 (3)	0.0530 (13)	
H6	0.8842	0.4053	−0.1121	0.064*	
C7	0.7826 (6)	0.4679 (2)	−0.0164 (3)	0.0453 (12)	
H7	0.8076	0.5198	−0.0365	0.054*	
C8	0.4677 (5)	0.6152 (2)	0.2817 (3)	0.0347 (10)	
C9	0.4411 (5)	0.7033 (2)	0.3016 (3)	0.0330 (10)	
C10	0.3652 (5)	0.7305 (2)	0.3723 (3)	0.0382 (11)	
H10	0.3305	0.6934	0.4131	0.046*	
C11	0.3407 (6)	0.8137 (2)	0.3823 (3)	0.0464 (12)	
H11	0.2869	0.8332	0.4290	0.056*	
C12	0.3965 (6)	0.8670 (2)	0.3226 (3)	0.0474 (12)	
H12	0.3790	0.9232	0.3275	0.057*	
C13	0.4786 (6)	0.8367 (2)	0.2553 (3)	0.0441 (12)	
H13	0.5204	0.8735	0.2166	0.053*	
C14	0.4049 (5)	0.5474 (2)	0.3330 (3)	0.0323 (10)	
C15	0.2398 (5)	0.5486 (2)	0.3512 (3)	0.0410 (11)	
H15	0.1692	0.5914	0.3300	0.049*	
C16	0.1819 (6)	0.4863 (2)	0.4004 (3)	0.0466 (12)	
H16	0.0725	0.4878	0.4133	0.056*	
C17	0.2839 (6)	0.4219 (3)	0.4306 (3)	0.0530 (13)	
H17	0.2435	0.3798	0.4634	0.064*	
C18	0.4474 (6)	0.4201 (3)	0.4120 (3)	0.0520 (13)	
H18	0.5165	0.3763	0.4319	0.062*	
C19	0.5076 (5)	0.4825 (2)	0.3643 (3)	0.0428 (11)	
H19	0.6180	0.4814	0.3529	0.051*	

### Atomic displacement parameters (Å^2^)

**Table:**

	*U*^11^	*U*^22^	*U*^33^	*U*^12^	*U*^13^	*U*^23^
Cu1	0.0471 (3)	0.0269 (3)	0.0353 (3)	0.0016 (2)	0.0168 (3)	0.0001 (2)
Br1	0.0552 (3)	0.0367 (2)	0.0453 (3)	0.0031 (2)	0.0238 (2)	0.0088 (2)
Br2	0.0575 (3)	0.0528 (3)	0.0716 (4)	0.0197 (2)	−0.0113 (3)	−0.0204 (3)
N1	0.057 (2)	0.0228 (17)	0.042 (2)	0.0029 (15)	0.0223 (19)	−0.0013 (15)
N2	0.048 (2)	0.0262 (18)	0.033 (2)	0.0023 (15)	0.0149 (18)	−0.0039 (15)
N3	0.041 (2)	0.0322 (19)	0.033 (2)	0.0046 (15)	0.0129 (17)	0.0022 (15)
O1	0.060 (2)	0.0265 (15)	0.0343 (18)	−0.0017 (13)	0.0203 (15)	−0.0002 (12)
O2	0.086 (3)	0.0286 (16)	0.068 (2)	−0.0033 (15)	0.038 (2)	0.0041 (15)
C1	0.038 (3)	0.034 (2)	0.031 (3)	0.0028 (18)	0.010 (2)	0.0000 (19)
C2	0.039 (3)	0.025 (2)	0.038 (3)	−0.0001 (17)	0.007 (2)	−0.0042 (18)
C3	0.042 (3)	0.031 (2)	0.046 (3)	−0.0022 (19)	0.009 (2)	−0.004 (2)
C4	0.053 (3)	0.026 (2)	0.066 (3)	−0.001 (2)	0.008 (3)	−0.004 (2)
C5	0.069 (4)	0.038 (3)	0.049 (3)	0.010 (2)	0.007 (3)	−0.014 (2)
C6	0.075 (4)	0.045 (3)	0.043 (3)	0.008 (2)	0.021 (3)	−0.009 (2)
C7	0.062 (3)	0.033 (2)	0.044 (3)	0.001 (2)	0.016 (3)	0.004 (2)
C8	0.034 (2)	0.034 (2)	0.036 (3)	−0.0020 (18)	0.005 (2)	−0.0017 (19)
C9	0.038 (3)	0.032 (2)	0.030 (2)	0.0024 (18)	0.009 (2)	0.0025 (18)
C10	0.049 (3)	0.036 (2)	0.033 (3)	−0.0011 (19)	0.016 (2)	−0.0008 (19)
C11	0.058 (3)	0.046 (3)	0.039 (3)	0.006 (2)	0.020 (2)	−0.006 (2)
C12	0.073 (4)	0.031 (2)	0.040 (3)	0.008 (2)	0.016 (3)	−0.002 (2)
C13	0.065 (3)	0.028 (2)	0.042 (3)	0.007 (2)	0.016 (3)	0.003 (2)
C14	0.040 (3)	0.030 (2)	0.029 (2)	0.0015 (18)	0.009 (2)	0.0044 (18)
C15	0.040 (3)	0.034 (2)	0.050 (3)	0.0055 (19)	0.009 (2)	0.001 (2)
C16	0.041 (3)	0.042 (3)	0.060 (3)	−0.009 (2)	0.020 (3)	−0.003 (2)
C17	0.071 (4)	0.039 (3)	0.052 (3)	−0.011 (2)	0.019 (3)	0.009 (2)
C18	0.066 (3)	0.037 (3)	0.054 (3)	0.011 (2)	0.014 (3)	0.015 (2)
C19	0.041 (3)	0.045 (3)	0.045 (3)	0.005 (2)	0.012 (2)	0.006 (2)

### Geometric parameters (Å, °)

**Table:**

Cu1—N2	1.966 (3)	C6—H6	0.9300
Cu1—N3	2.018 (3)	C7—H7	0.9300
Cu1—O1	2.083 (2)	C8—C14	1.474 (5)
Cu1—Br1	2.3469 (6)	C8—C9	1.490 (5)
Cu1—Br2	2.5931 (8)	C9—C10	1.370 (5)
N1—C1	1.355 (5)	C10—C11	1.381 (5)
N1—N2	1.358 (4)	C10—H10	0.9300
N1—H1	0.8600	C11—C12	1.369 (6)
N2—C8	1.304 (5)	C11—H11	0.9300
N3—C13	1.338 (5)	C12—C13	1.373 (5)
N3—C9	1.363 (5)	C12—H12	0.9300
O1—C1	1.242 (4)	C13—H13	0.9300
O2—C3	1.361 (5)	C14—C19	1.388 (5)
O2—H2	0.8200	C14—C15	1.397 (5)
C1—C2	1.471 (5)	C15—C16	1.375 (5)
C2—C7	1.393 (5)	C15—H15	0.9300
C2—C3	1.396 (5)	C16—C17	1.374 (6)
C3—C4	1.388 (5)	C16—H16	0.9300
C4—C5	1.373 (6)	C17—C18	1.386 (6)
C4—H4	0.9300	C17—H17	0.9300
C5—C6	1.373 (6)	C18—C19	1.371 (5)
C5—H5	0.9300	C18—H18	0.9300
C6—C7	1.383 (5)	C19—H19	0.9300
			
N2—Cu1—N3	78.67 (12)	C6—C7—C2	121.9 (4)
N2—Cu1—O1	77.25 (11)	C6—C7—H7	119.0
N3—Cu1—O1	153.13 (12)	C2—C7—H7	119.0
N2—Cu1—Br1	159.76 (10)	N2—C8—C14	125.3 (3)
N3—Cu1—Br1	98.37 (9)	N2—C8—C9	111.4 (3)
O1—Cu1—Br1	100.10 (7)	C14—C8—C9	123.3 (3)
N2—Cu1—Br2	95.15 (10)	N3—C9—C10	121.8 (3)
N3—Cu1—Br2	100.15 (10)	N3—C9—C8	114.2 (3)
O1—Cu1—Br2	93.71 (8)	C10—C9—C8	124.0 (3)
Br1—Cu1—Br2	105.07 (2)	C9—C10—C11	119.2 (4)
C1—N1—N2	114.6 (3)	C9—C10—H10	120.4
C1—N1—H1	122.7	C11—C10—H10	120.4
N2—N1—H1	122.7	C12—C11—C10	119.2 (4)
C8—N2—N1	124.2 (3)	C12—C11—H11	120.4
C8—N2—Cu1	120.7 (2)	C10—C11—H11	120.4
N1—N2—Cu1	115.1 (2)	C11—C12—C13	119.2 (4)
C13—N3—C9	118.0 (3)	C11—C12—H12	120.4
C13—N3—Cu1	127.2 (3)	C13—C12—H12	120.4
C9—N3—Cu1	114.6 (2)	N3—C13—C12	122.6 (4)
C1—O1—Cu1	113.3 (2)	N3—C13—H13	118.7
C3—O2—H2	109.5	C12—C13—H13	118.7
O1—C1—N1	119.1 (3)	C19—C14—C15	119.2 (3)
O1—C1—C2	122.9 (4)	C19—C14—C8	121.1 (4)
N1—C1—C2	118.0 (3)	C15—C14—C8	119.7 (3)
C7—C2—C3	118.3 (4)	C16—C15—C14	119.8 (4)
C7—C2—C1	116.9 (3)	C16—C15—H15	120.1
C3—C2—C1	124.9 (4)	C14—C15—H15	120.1
O2—C3—C4	121.6 (4)	C17—C16—C15	120.7 (4)
O2—C3—C2	118.4 (3)	C17—C16—H16	119.7
C4—C3—C2	120.0 (4)	C15—C16—H16	119.7
C5—C4—C3	119.7 (4)	C16—C17—C18	119.7 (4)
C5—C4—H4	120.1	C16—C17—H17	120.1
C3—C4—H4	120.1	C18—C17—H17	120.1
C4—C5—C6	121.9 (4)	C19—C18—C17	120.2 (4)
C4—C5—H5	119.1	C19—C18—H18	119.9
C6—C5—H5	119.1	C17—C18—H18	119.9
C5—C6—C7	118.2 (4)	C18—C19—C14	120.4 (4)
C5—C6—H6	120.9	C18—C19—H19	119.8
C7—C6—H6	120.9	C14—C19—H19	119.8
			
C1—N1—N2—C8	172.6 (4)	C3—C4—C5—C6	1.5 (8)
C1—N1—N2—Cu1	−6.5 (4)	C4—C5—C6—C7	−1.7 (8)
N3—Cu1—N2—C8	−4.6 (3)	C5—C6—C7—C2	0.7 (7)
O1—Cu1—N2—C8	−172.6 (3)	C3—C2—C7—C6	0.5 (7)
Br1—Cu1—N2—C8	−88.0 (4)	C1—C2—C7—C6	179.9 (4)
Br2—Cu1—N2—C8	94.8 (3)	N1—N2—C8—C14	2.8 (6)
N3—Cu1—N2—N1	174.6 (3)	Cu1—N2—C8—C14	−178.1 (3)
O1—Cu1—N2—N1	6.6 (3)	N1—N2—C8—C9	−176.9 (4)
Br1—Cu1—N2—N1	91.2 (4)	Cu1—N2—C8—C9	2.2 (5)
Br2—Cu1—N2—N1	−86.1 (3)	C13—N3—C9—C10	−2.2 (6)
N2—Cu1—N3—C13	−179.8 (4)	Cu1—N3—C9—C10	172.6 (3)
O1—Cu1—N3—C13	−153.1 (3)	C13—N3—C9—C8	178.5 (4)
Br1—Cu1—N3—C13	−20.1 (4)	Cu1—N3—C9—C8	−6.8 (4)
Br2—Cu1—N3—C13	87.0 (3)	N2—C8—C9—N3	3.1 (5)
N2—Cu1—N3—C9	6.0 (3)	C14—C8—C9—N3	−176.6 (4)
O1—Cu1—N3—C9	32.7 (5)	N2—C8—C9—C10	−176.2 (4)
Br1—Cu1—N3—C9	165.7 (3)	C14—C8—C9—C10	4.1 (7)
Br2—Cu1—N3—C9	−87.2 (3)	N3—C9—C10—C11	3.4 (7)
N2—Cu1—O1—C1	−6.0 (3)	C8—C9—C10—C11	−177.3 (4)
N3—Cu1—O1—C1	−32.8 (5)	C9—C10—C11—C12	−1.6 (7)
Br1—Cu1—O1—C1	−165.6 (3)	C10—C11—C12—C13	−1.4 (7)
Br2—Cu1—O1—C1	88.4 (3)	C9—N3—C13—C12	−0.9 (6)
Cu1—O1—C1—N1	4.5 (5)	Cu1—N3—C13—C12	−174.9 (3)
Cu1—O1—C1—C2	−174.8 (3)	C11—C12—C13—N3	2.7 (7)
N2—N1—C1—O1	1.1 (6)	N2—C8—C14—C19	48.2 (6)
N2—N1—C1—C2	−179.6 (3)	C9—C8—C14—C19	−132.1 (4)
O1—C1—C2—C7	3.1 (6)	N2—C8—C14—C15	−132.3 (4)
N1—C1—C2—C7	−176.1 (4)	C9—C8—C14—C15	47.4 (6)
O1—C1—C2—C3	−177.5 (4)	C19—C14—C15—C16	0.7 (6)
N1—C1—C2—C3	3.2 (6)	C8—C14—C15—C16	−178.8 (4)
C7—C2—C3—O2	179.6 (4)	C14—C15—C16—C17	−1.2 (7)
C1—C2—C3—O2	0.3 (6)	C15—C16—C17—C18	0.5 (7)
C7—C2—C3—C4	−0.7 (7)	C16—C17—C18—C19	0.6 (7)
C1—C2—C3—C4	180.0 (4)	C17—C18—C19—C14	−1.1 (7)
O2—C3—C4—C5	179.5 (4)	C15—C14—C19—C18	0.4 (7)
C2—C3—C4—C5	−0.2 (7)	C8—C14—C19—C18	179.9 (4)

### Hydrogen-bond geometry (Å, °)

**Table:**

*D*—H···*A*	*D*—H	H···*A*	*D*···*A*	*D*—H···*A*
N1—H1···O2	0.86	1.92	2.574 (4)	131
O2—H2···Br2^i^	0.82	2.35	3.153 (3)	166
C11—H11···O1^ii^	0.93	2.58	3.503 (5)	170
C10—H10···Br1^ii^	0.93	2.81	3.575 (4)	141
C15—H15···Br2^iii^	0.93	2.82	3.742 (4)	171

Symmetry codes: (i) −*x*+3/2, *y*−1/2, −*z*+1/2; (ii) *x*−1/2, −*y*+3/2, *z*+1/2; (iii) *x*−1, *y*, *z*.
